# Balanced cortical microcircuitry for maintaining short-term memory

**DOI:** 10.1186/1471-2202-13-S1-O11

**Published:** 2012-07-16

**Authors:** Sukbin Lim, Mark S Goldman

**Affiliations:** 1 Center for Neuroscience, University of California, Davis, Davis, CA 96518, USA; 2 Department of Neurobiology, Physiology, and Behavior, University of California, Davis, Davis, CA 96518, USA; 3 Department of Ophthalmology and Visual Sciences, University of California, Davis, Davis, CA 96518, USA

## 

Persistent patterns of neural activity that last long after the offset of a stimulus are thought to be the neural substrate for short-term memory. Because the observed decay of persistent activity in memory circuits is much slower than the typical decay time constants associated with synaptic or intrinsic neuronal dynamics, it has been suggested that network interactions must be used to prolong the duration of persistent activity. Most often, these network interactions have been assumed to mediate positive feedback between neurons that supports a long-lasting reverberation of activity. However, most positive feedback models do not naturally fit the architecture of working memory-storing structures in neocortex that have been suggested to exhibit a close balance between excitation and inhibition. Furthermore, positive feedback models of analog memory storage are highly non-robust against commonly studied perturbations in network connectivity.

Here, we suggest a complementary mechanism for generating persistent activity based on the principle of corrective negative-feedback: an error-correcting signal of the form of a time-derivative of activity reduces memory slip when it occurs. Using analytic calculations, we show that neocortical circuit models with the observed balance in strength, but with different kinetics, between excitatory and inhibitory synaptic inputs, produce a negative-derivative feedback signal that counteracts drifts in persistent activity. The networks maintain a continuum of stable firing rates even in the presence of intrinsic input-output nonlinearity, while still remaining responsive to external memory inputs. More generally, the networks act as temporal integrators of their inputs, for example converting step-like input into linearly ramping activity.

Memory networks operating in this balanced regime are robust against many commonly studied perturbations to synaptic weights that grossly disrupt the performance of persistent activity circuits based on positive feedback. Specifically, in response to uniform changes in synaptic excitation, synaptic inhibition, intrinsic neuronal gains or loss of a fraction of excitatory or inhibitory neurons, there is minimal decay or instability in persistent firing. Furthermore, spiking network models implementing derivative feedback generate persistent firing with Poisson-like statistics, as has been observed experimentally. This observed highly irregular activity occurs across a graded range of firing rates, and arises because the close balance between excitation and inhibition results in spikes being triggered primarily by fluctuations in, rather than means levels of, synaptic inputs.

To generate experimental predictions that distinguish among different mechanisms for short-term memory, we compared the correlation structure of excitatory and inhibitory inputs in the negative-derivative feedback models to that of typical analog memory models based on positive feedback. Negative-derivative feedback models exhibit a strong positive correlation between inhibitory and excitatory synaptic inputs, as suggested by recent experiments. By contrast, similarly responding neurons in positive feedback models either exhibited anti-correlations or weak correlations between their excitatory and inhibitory inputs.

Altogether, this work suggests a new paradigm for short-term memory storage based upon a balanced network with cortical-like architecture. Stabilization of responses through negative feedback is a common principle in engineering control systems. Our work suggests that a similar principle might be inherent to the circuitry of working memory systems.

